# Methyl 2-(5-iodo-7-methyl-3-methyl­sulfinyl-1-benzofuran-2-yl)acetate

**DOI:** 10.1107/S1600536809016298

**Published:** 2009-05-14

**Authors:** Hong Dae Choi, Pil Ja Seo, Byeng Wha Son, Uk Lee

**Affiliations:** a Department of Chemistry, Dongeui University, San 24 Kaya-dong Busanjin-gu, Busan 614-714, Republic of Korea; b Department of Chemistry, Pukyong National University, 599-1 Daeyeon 3-dong Nam-gu, Busan 608-737, Republic of Korea

## Abstract

There are two symmetry-independent mol­ecules in the asymmetric unit of the title compound, C_13_H_13_IO_4_S. In each mol­ecule, the O atom and the methyl group of the methyl­sulfinyl substituent lie on opposite sides of the plane of the benzofuran fragment. The crystal structure is stabilized by aromatic π–π inter­actions between the benzene and furan ring [centroid–centroid distance = 3.866 (7) Å], and by inter­molecular C—H⋯π inter­actions and a sulfin­yl–sulfinyl inter­action [S⋯O = 3.025 (4) Å]. The crystal structure also exhibits weak inter­molecular C—H⋯O hydrogen bonds and two different I⋯O halogen bonds.

## Related literature

For the crystal structures of similar alkyl 2-(5-iodo-3-methyl­sulfinyl-1-benzofuran-2-yl)acetate derivatives. see: Choi *et al.* (2008**a* ▶,b* ▶). For halogen bonding, see: Politzer *et al.* (2007 ▶). For carbon­yl–carbonyl inter­actions, see: Allen *et al.* (1998 ▶).
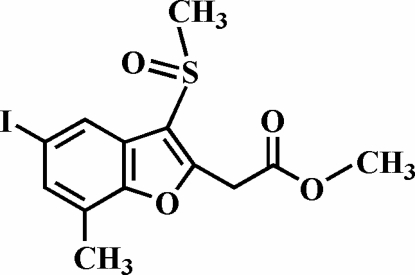


## Experimental

### 

#### Crystal data

C_13_H_13_IO_4_S
*M* *_r_* = 392.19
Triclinic, 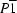

*a* = 7.5424 (4) Å
*b* = 11.2177 (6) Å
*c* = 17.845 (1) Å
α = 77.701 (1)°
β = 88.074 (1)°
γ = 88.229 (1)°
*V* = 1473.92 (14) Å^3^
*Z* = 4
Mo *K*α radiation
μ = 2.32 mm^−1^
*T* = 293 K
0.30 × 0.20 × 0.10 mm

#### Data collection

Bruker SMART CCD diffractometer
Absorption correction: multi-scan (*SADABS*; Sheldrick, 1999 ▶) *T*_min_ = 0.574, *T*_max_ = 0.790
12617 measured reflections
6248 independent reflections
5328 reflections with *I* > 2σ(*I*)
*R*_int_ = 0.027

#### Refinement

*R*[*F* ^2^ > 2σ(*F* ^2^)] = 0.050
*wR*(*F* ^2^) = 0.088
*S* = 1.18
6248 reflections
349 parameters
H-atom parameters constrained
Δρ_max_ = 1.15 e Å^−3^
Δρ_min_ = −1.46 e Å^−3^

### 

Data collection: *SMART* (Bruker, 2001 ▶); cell refinement: *SAINT* (Bruker, 2001 ▶); data reduction: *SAINT*; program(s) used to solve structure: *SHELXS97* (Sheldrick, 2008 ▶); program(s) used to refine structure: *SHELXL97* (Sheldrick, 2008 ▶); molecular graphics: *ORTEP-3* (Farrugia, 1997 ▶) and *DIAMOND* (Brandenburg, 1998 ▶); software used to prepare material for publication: *SHELXL97*.

## Supplementary Material

Crystal structure: contains datablocks global, I. DOI: 10.1107/S1600536809016298/gw2062sup1.cif

Structure factors: contains datablocks I. DOI: 10.1107/S1600536809016298/gw2062Isup2.hkl

Additional supplementary materials:  crystallographic information; 3D view; checkCIF report

## Figures and Tables

**Table 1:** Selected interatomic distances (Å)

I1⋯O7^i^	3.300 (3)
I2⋯O3^ii^	3.264 (3)

Symmetry codes: (i) 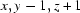; (ii) 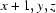.

**Table 2:** Hydrogen-bond geometry (Å, °)

*D*—H⋯*A*	*D*—H	H⋯*A*	*D*⋯*A*	*D*—H⋯*A*
C5—H5⋯O3^iii^	0.93	2.59	3.430 (6)	150
C11—H11*B*⋯O8	0.96	2.51	3.254 (6)	134
C12—H12*C*⋯O4^iv^	0.96	2.58	3.489 (6)	158
C16—H16⋯O8^v^	0.93	2.48	3.373 (6)	160
C18—H18⋯O4^vi^	0.93	2.44	3.297 (6)	153
C22—H22*B*⋯O8^vii^	0.97	2.27	3.215 (6)	164
C12—H12*A*⋯*Cg*1^iii^	0.96	2.85	3.734 (7)	153
C24—H24*A*⋯*Cg*3^viii^	0.96	2.73	3.574 (7)	147

Symmetry codes: (iii) 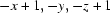; (iv) 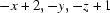; (v) 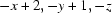; (vi) 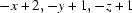; (vii) 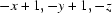; (viii) 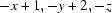. *Cg*1 and *Cg*3 are the centroids of the C2–C7 benzene ring and the C14/C15/C20/O5/C21 furan ring, respectively.

**Fig. 1.**
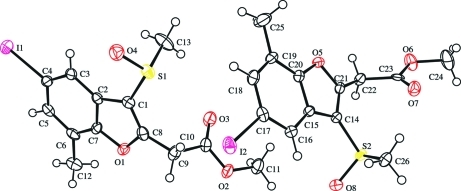
The molecular structure of the title compound, showing displacement ellipsoids drawn at the 30% probability level.

**Fig. 2.**
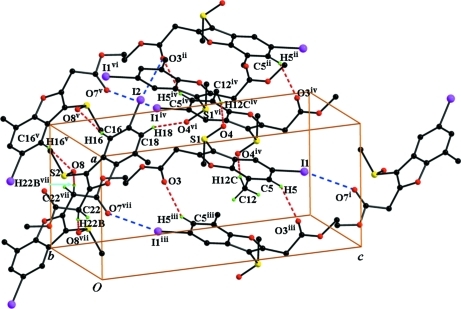
C—H···O hydrogen bond and I···O halogen bond (dotted lines) in the title compound [Symmetry code: (i) x, y - 1, z + 1; (ii) x + 1, y, z; (iii) -x + 1, -y, -z + 1; (iv) -x + 2, -y, -z + 1; (v) -x + 2, -y + 1, -z; (vi) 2 - x, 1 - y, 1 - z; (vii) - x + 1, - y + 1, - z.]

**Fig. 3.**
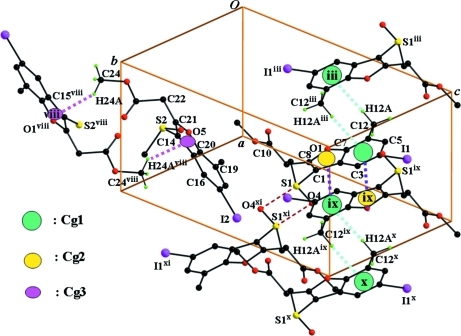
π—π, C—H···π, and S···O interactions (dotted lines) in the title compound. Cg denotes the ring centroids [Symmetry code: (iii) - x + 1, - y, - z + 1; (viii) - x + 1, - y + 2, - z; (ix) - x + 2, - y, - z + 1; (x) x + 1, y, z; (xi) - x + 2, - y + 1, - z + 1.]

## supplementary crystallographic information

### Comment

This work is related to our previous communications on the synthesis and structure of alkyl 2-(5-iodo-3-methylsulfinyl-1-benzofuran-2-yl)acetate analogues, *viz*. isopropyl 2-(5-iodo-3-methylsulfinyl-1-benzofuran-2-yl)acetate (Choi *et al.*, 2008*a*) and isopropyl 2-(5-iodo-7-methyl-3-methylsulfinyl-1-benzofuran-2-yl)acetate (Choi *et al.*, 2008*b*). Here we report the crystal structure of the title compound, methyl 2-(5-iodo-7-methyl-3-methylsulfinyl-1-benzofuran-2-yl)acetate (Fig. 1). The benzofuran unit is essentially planar, with a mean deviation of 0.017 (4) Å for two independent molecules, from the least-squares plane defined by the nine constituent atoms.

The molecular packing (Fig. 2 & 3) is stabilized by aromatic π—π interactions between the benzene ring and the furan ring of adjacent benzofuran units, with a *Cg*1···*Cg*2^ix^ distance of 3.866 (7) Å (*Cg*1 and *Cg*2 are the centroids of the C2–C7 benzene ring and the C1/C2/C7/O1/C8 furan ring; symmetry code as in Fig. 3). The crystal packing is further stabilized by C—H···π interactions (Table 2 and Fig. 3); the first between the methyl H atom and the benzene ring of the benzofuran unit, *i.e.* C12—H12A···*Cg*1^iii^, the second between the methyl H atom and the furan ring of the benzofuran unit, *i.e.* C24—H24A···*Cg*3^viii^, respectively (*Cg*3 is the centroid of the C14/C15/C20/O5/C21 furan ring; symmetry code as in Fig. 3), and by an intermolecular sulfinyl–sulfinyl interaction interpreted as simliar to a type–II carbonyl–carbonyl interaction (Allen *et al.*, 1998), with S1···O4^xi^ and O4···S1^xi^ distance of 3.025 (4) Å (Fig. 3; symmetry code as in Fig. 3). In addition, the crystal packing exhibits weak intermolecular C—H···O hydrogen bonds (Fig. 2 and Table 2). In crystal structure, there are two different I···O halogen bonds (Politzer *et al.*, 2007) between the two independent iodine atoms and the oxygen atoms of neighbouring C═O units (Fig. 2 and Table 1).

### Experimental

77% 3–chloroperoxybenzoic acid (123 mg, 0.55 mmol) was added in small portions to a stirred solution of methyl 2-(5-iodo-7-methyl-3-methylsulfanyl-1-benzofuran-2-yl)acetate (202 mg, 0.5 mmol) in dichloromethane (30 ml) at 273 K. After being stirred for 3 h at room temperature, the mixture was washed with saturated sodium bicarbonate solution and the organic layer was separated, dried over magnesium sulfate, filtered and concentrated in vacuum. The residue was purified by column chromatography (ethyl acetate) to afford the title compound as a colorless solid [yield 83%, m.p. 414–415 K; *R*_f_ = 0.63 (ethyl acetate)]. Single crystals suitable for X-ray diffraction were prepared by evaporation of a solution of the title compound in benzene at room temperature. Spectroscopic analysis: ^1^H NMR (CDCl_3_, 400 MHz) δ 2.46 (s, 3H), 3.05 (s, 3H), 3.76 (s, 3H), 4.07 (s, 2H), 7.49 (s, 1H), 8.07 (s, 1H); EI—MS 392 [*M*^+^].

### Refinement

All H atoms were geometrically positioned and refined using a riding model, with C—H = 0.93 Å for the aryl, 0.97 Å for the methylene, and 0.96 Å for the methyl H atoms. *U*_iso_(H) = 1.2Ueq(C) for the aryl and methylene H atoms, and 1.5Ueq(C) for methyl H atoms.

### Crystal data

**Table:**

C_13_H_13_IO_4_S	*Z* = 4
*M**_r_* = 392.19	*F*(000) = 768
Triclinic, *P*1	*D*_x_ = 1.767 Mg m^−^^3^
Hall symbol: -p 1	Mo *K*α radiation, λ = 0.71073 Å
*a* = 7.5424 (4) Å	Cell parameters from 6307 reflections
*b* = 11.2177 (6) Å	θ = 2.3–28.2°
*c* = 17.845 (1) Å	µ = 2.32 mm^−^^1^
α = 77.701 (1)°	*T* = 293 K
β = 88.074 (1)°	Block, colorless
γ = 88.229 (1)°	0.30 × 0.20 × 0.10 mm
*V* = 1473.92 (14) Å^3^	

### Data collection

**Table:**

Bruker SMART CCD diffractometer	6248 independent reflections
Radiation source: fine-focus sealed tube	5328 reflections with *I* > 2σ(*I*)
graphite	*R*_int_ = 0.027
Detector resolution: 10.0 pixels mm^-1^	θ_max_ = 27.0°, θ_min_ = 1.9°
φ and ω scans	*h* = −9→9
Absorption correction: multi-scan (*SADABS*; Sheldrick, 1999)	*k* = −14→14
*T*_min_ = 0.574, *T*_max_ = 0.790	*l* = −22→22
12617 measured reflections	

### Refinement

**Table:**

Refinement on *F*^2^	Primary atom site location: structure-invariant direct methods
Least-squares matrix: full	Secondary atom site location: difference Fourier map
*R*[*F*^2^ > 2σ(*F*^2^)] = 0.050	Hydrogen site location: difference Fourier map
*wR*(*F*^2^) = 0.088	H-atom parameters constrained
*S* = 1.18	*w* = 1/[σ^2^(*F*_o_^2^) + (0.0184*P*)^2^ + 4.3135*P*] where *P* = (*F*_o_^2^ + 2*F*_c_^2^)/3
6248 reflections	(Δ/σ)_max_ < 0.001
349 parameters	Δρ_max_ = 1.15 e Å^−^^3^
0 restraints	Δρ_min_ = −1.46 e Å^−^^3^

### Special details

**Table:**

Geometry. All e.s.d.'s (except the e.s.d. in the dihedral angle between two l.s. planes) are estimated using the full covariance matrix. The cell e.s.d.'s are taken into account individually in the estimation of e.s.d.'s in distances, angles and torsion angles; correlations between e.s.d.'s in cell parameters are only used when they are defined by crystal symmetry. An approximate (isotropic) treatment of cell e.s.d.'s is used for estimating e.s.d.'s involving l.s. planes.
Refinement. Refinement of *F*^2^ against ALL reflections. The weighted *R*-factor *wR* and goodness of fit *S* are based on *F*^2^, conventional *R*-factors *R* are based on *F*, with *F* set to zero for negative *F*^2^. The threshold expression of *F*^2^ > σ(*F*^2^) is used only for calculating *R*-factors(gt) *etc*. and is not relevant to the choice of reflections for refinement. *R*-factors based on *F*^2^ are statistically about twice as large as those based on *F*, and *R*- factors based on ALL data will be even larger.

### Fractional atomic coordinates and isotropic or equivalent isotropic displacement parameters (Å^2^)

**Table:**

	*x*	*y*	*z*	*U*_iso_*/*U*_eq_	
I1	0.66948 (5)	−0.00958 (3)	0.780861 (19)	0.03974 (11)	
S1	0.92033 (15)	0.36686 (11)	0.47095 (7)	0.0253 (3)	
O1	0.7992 (4)	0.0463 (3)	0.43207 (17)	0.0234 (7)	
O2	0.7940 (4)	0.2574 (3)	0.20886 (18)	0.0285 (8)	
O3	0.6320 (5)	0.3131 (3)	0.30365 (19)	0.0340 (8)	
O4	1.0056 (5)	0.3653 (3)	0.5448 (2)	0.0343 (8)	
C1	0.8593 (6)	0.2142 (4)	0.4747 (3)	0.0210 (9)	
C2	0.7987 (6)	0.1252 (4)	0.5400 (3)	0.0193 (9)	
C3	0.7743 (6)	0.1181 (4)	0.6187 (3)	0.0217 (10)	
H3	0.8012	0.1827	0.6411	0.026*	
C4	0.7085 (6)	0.0108 (4)	0.6616 (3)	0.0226 (10)	
C5	0.6671 (6)	−0.0880 (4)	0.6299 (3)	0.0249 (10)	
H5	0.6212	−0.1579	0.6614	0.030*	
C6	0.6938 (6)	−0.0833 (4)	0.5515 (3)	0.0260 (10)	
C7	0.7615 (5)	0.0234 (4)	0.5097 (2)	0.0192 (9)	
C8	0.8613 (6)	0.1634 (4)	0.4126 (3)	0.0236 (10)	
C9	0.9133 (6)	0.2074 (5)	0.3309 (3)	0.0304 (12)	
H9A	0.9629	0.1393	0.3109	0.037*	
H9B	1.0056	0.2669	0.3274	0.037*	
C10	0.7613 (6)	0.2650 (4)	0.2812 (3)	0.0229 (10)	
C11	0.6621 (7)	0.3127 (5)	0.1547 (3)	0.0333 (12)	
H11A	0.5579	0.2640	0.1625	0.050*	
H11B	0.7086	0.3177	0.1034	0.050*	
H11C	0.6319	0.3933	0.1621	0.050*	
C12	0.6516 (7)	−0.1891 (5)	0.5159 (3)	0.0331 (12)	
H12A	0.5628	−0.1639	0.4781	0.050*	
H12B	0.6079	−0.2550	0.5550	0.050*	
H12C	0.7571	−0.2158	0.4920	0.050*	
C13	0.7001 (7)	0.4299 (5)	0.4797 (4)	0.0403 (14)	
H13A	0.6444	0.3883	0.5267	0.060*	
H13B	0.6317	0.4198	0.4371	0.060*	
H13C	0.7069	0.5151	0.4798	0.060*	
I2	1.27204 (4)	0.47039 (3)	0.246411 (19)	0.02932 (10)	
S2	0.68848 (15)	0.62050 (11)	−0.02104 (6)	0.0208 (2)	
O5	0.5483 (4)	0.7323 (3)	0.16778 (17)	0.0228 (7)	
O6	0.2057 (4)	0.9129 (3)	−0.0229 (2)	0.0348 (9)	
O7	0.5006 (4)	0.8964 (3)	−0.04426 (19)	0.0307 (8)	
O8	0.7522 (4)	0.4897 (3)	−0.01091 (18)	0.0288 (8)	
C14	0.6717 (6)	0.6561 (4)	0.0710 (2)	0.0192 (9)	
C15	0.7950 (6)	0.6310 (4)	0.1331 (2)	0.0201 (9)	
C16	0.9597 (6)	0.5710 (4)	0.1474 (3)	0.0226 (10)	
H16	1.0203	0.5374	0.1103	0.027*	
C17	1.0283 (6)	0.5640 (4)	0.2189 (3)	0.0241 (10)	
C18	0.9410 (7)	0.6153 (4)	0.2756 (3)	0.0287 (11)	
H18	0.9940	0.6092	0.3227	0.034*	
C19	0.7787 (7)	0.6744 (5)	0.2632 (3)	0.0291 (11)	
C20	0.7105 (6)	0.6795 (4)	0.1915 (3)	0.0221 (10)	
C21	0.5275 (6)	0.7141 (4)	0.0946 (2)	0.0189 (9)	
C22	0.3549 (6)	0.7575 (4)	0.0601 (3)	0.0232 (10)	
H22A	0.2778	0.7819	0.0991	0.028*	
H22B	0.3000	0.6902	0.0442	0.028*	
C23	0.3682 (6)	0.8633 (4)	−0.0081 (3)	0.0215 (10)	
C24	0.1942 (8)	1.0119 (5)	−0.0898 (3)	0.0457 (15)	
H24A	0.2691	1.0767	−0.0837	0.069*	
H24B	0.0737	1.0418	−0.0953	0.069*	
H24C	0.2322	0.9828	−0.1347	0.069*	
C25	0.6774 (8)	0.7295 (6)	0.3224 (3)	0.0482 (16)	
H25A	0.7436	0.7163	0.3686	0.072*	
H25B	0.5643	0.6917	0.3333	0.072*	
H25C	0.6600	0.8155	0.3031	0.072*	
C26	0.8772 (6)	0.7101 (5)	−0.0563 (3)	0.0284 (11)	
H26A	0.9763	0.6818	−0.0243	0.043*	
H26B	0.8507	0.7941	−0.0556	0.043*	
H26C	0.9061	0.7024	−0.1079	0.043*	

### Atomic displacement parameters (Å^2^)

**Table:**

	*U*^11^	*U*^22^	*U*^33^	*U*^12^	*U*^13^	*U*^23^
I1	0.0579 (3)	0.0384 (2)	0.02000 (17)	0.01037 (17)	0.00379 (15)	−0.00193 (14)
S1	0.0221 (6)	0.0216 (6)	0.0315 (6)	−0.0007 (5)	−0.0032 (5)	−0.0038 (5)
O1	0.0225 (16)	0.0297 (18)	0.0195 (16)	0.0001 (14)	−0.0032 (13)	−0.0085 (14)
O2	0.0289 (18)	0.038 (2)	0.0172 (16)	0.0048 (15)	−0.0014 (14)	−0.0026 (14)
O3	0.0275 (19)	0.045 (2)	0.0254 (18)	0.0095 (16)	0.0018 (15)	−0.0014 (16)
O4	0.040 (2)	0.030 (2)	0.0317 (19)	−0.0043 (16)	−0.0048 (16)	−0.0033 (16)
C1	0.019 (2)	0.020 (2)	0.024 (2)	−0.0017 (18)	−0.0020 (18)	−0.0055 (19)
C2	0.016 (2)	0.017 (2)	0.024 (2)	0.0020 (17)	−0.0005 (17)	−0.0028 (18)
C3	0.024 (2)	0.021 (2)	0.021 (2)	0.0042 (18)	−0.0073 (18)	−0.0058 (18)
C4	0.020 (2)	0.029 (3)	0.020 (2)	0.0078 (19)	−0.0036 (18)	−0.0077 (19)
C5	0.022 (2)	0.018 (2)	0.032 (3)	0.0036 (18)	0.0000 (19)	−0.0002 (19)
C6	0.018 (2)	0.023 (2)	0.038 (3)	0.0060 (19)	−0.005 (2)	−0.009 (2)
C7	0.014 (2)	0.028 (2)	0.016 (2)	0.0004 (18)	−0.0050 (16)	−0.0048 (18)
C8	0.016 (2)	0.025 (2)	0.028 (2)	0.0031 (18)	−0.0052 (18)	−0.001 (2)
C9	0.021 (2)	0.048 (3)	0.020 (2)	0.005 (2)	0.0014 (19)	−0.002 (2)
C10	0.018 (2)	0.027 (3)	0.021 (2)	−0.0007 (19)	−0.0011 (18)	0.0006 (19)
C11	0.032 (3)	0.044 (3)	0.021 (2)	0.001 (2)	−0.005 (2)	−0.002 (2)
C12	0.026 (3)	0.028 (3)	0.048 (3)	0.001 (2)	−0.004 (2)	−0.015 (2)
C13	0.022 (3)	0.023 (3)	0.075 (4)	0.002 (2)	−0.007 (3)	−0.010 (3)
I2	0.02201 (16)	0.03174 (19)	0.03059 (18)	0.00357 (13)	−0.00415 (13)	0.00141 (14)
S2	0.0241 (6)	0.0247 (6)	0.0145 (5)	0.0012 (5)	−0.0013 (4)	−0.0061 (4)
O5	0.0260 (17)	0.0245 (17)	0.0187 (16)	0.0080 (13)	−0.0023 (13)	−0.0074 (13)
O6	0.0237 (18)	0.032 (2)	0.043 (2)	0.0059 (15)	−0.0063 (16)	0.0050 (16)
O7	0.0295 (19)	0.033 (2)	0.0262 (18)	0.0012 (15)	0.0048 (15)	0.0008 (15)
O8	0.037 (2)	0.0257 (18)	0.0240 (17)	0.0027 (15)	0.0050 (15)	−0.0080 (14)
C14	0.022 (2)	0.020 (2)	0.015 (2)	−0.0008 (18)	0.0016 (17)	−0.0042 (17)
C15	0.026 (2)	0.019 (2)	0.016 (2)	−0.0026 (18)	0.0017 (18)	−0.0044 (17)
C16	0.024 (2)	0.023 (2)	0.020 (2)	0.0008 (19)	0.0015 (18)	−0.0059 (19)
C17	0.022 (2)	0.020 (2)	0.027 (2)	0.0006 (19)	−0.0035 (19)	0.0011 (19)
C18	0.036 (3)	0.030 (3)	0.020 (2)	0.004 (2)	−0.011 (2)	−0.005 (2)
C19	0.041 (3)	0.031 (3)	0.016 (2)	0.010 (2)	−0.006 (2)	−0.009 (2)
C20	0.027 (2)	0.020 (2)	0.018 (2)	0.0035 (19)	−0.0024 (18)	−0.0028 (18)
C21	0.024 (2)	0.019 (2)	0.014 (2)	−0.0021 (18)	−0.0015 (17)	−0.0018 (17)
C22	0.022 (2)	0.030 (3)	0.017 (2)	−0.0006 (19)	0.0015 (18)	−0.0037 (19)
C23	0.027 (2)	0.015 (2)	0.026 (2)	0.0066 (19)	0.001 (2)	−0.0125 (19)
C24	0.037 (3)	0.038 (3)	0.053 (4)	0.011 (3)	−0.011 (3)	0.011 (3)
C25	0.057 (4)	0.066 (4)	0.027 (3)	0.029 (3)	−0.011 (3)	−0.023 (3)
C26	0.031 (3)	0.032 (3)	0.021 (2)	−0.004 (2)	0.008 (2)	−0.002 (2)

### Geometric parameters (Å, °)

**Table:**

I1—C4	2.103 (4)	I2—C17	2.108 (4)
I1—O7^i^	3.300 (3)	I2—O3^iii^	3.264 (3)
S1—O4	1.482 (4)	S2—O8	1.505 (3)
S1—C1	1.773 (5)	S2—C14	1.771 (4)
S1—C13	1.800 (5)	S2—C26	1.786 (5)
S1—O4^ii^	3.025 (4)	O5—C21	1.379 (5)
O1—C7	1.375 (5)	O5—C20	1.382 (5)
O1—C8	1.378 (6)	O6—C23	1.341 (5)
O2—C10	1.326 (6)	O6—C24	1.451 (6)
O2—C11	1.442 (6)	O7—C23	1.196 (5)
O3—C10	1.198 (5)	C14—C21	1.351 (6)
C1—C8	1.349 (6)	C14—C15	1.446 (6)
C1—C2	1.435 (6)	C15—C20	1.400 (6)
C2—C3	1.397 (6)	C15—C16	1.400 (6)
C2—C7	1.403 (6)	C16—C17	1.379 (6)
C3—C4	1.378 (6)	C16—H16	0.9300
C3—H3	0.9300	C17—C18	1.403 (7)
C4—C5	1.395 (7)	C18—C19	1.376 (7)
C5—C6	1.397 (7)	C18—H18	0.9300
C5—H5	0.9300	C19—C20	1.385 (6)
C6—C7	1.371 (7)	C19—C25	1.509 (7)
C6—C12	1.507 (7)	C21—C22	1.482 (6)
C8—C9	1.481 (6)	C22—C23	1.511 (6)
C9—C10	1.516 (6)	C22—H22A	0.9700
C9—H9A	0.9700	C22—H22B	0.9700
C9—H9B	0.9700	C24—H24A	0.9600
C11—H11A	0.9600	C24—H24B	0.9600
C11—H11B	0.9600	C24—H24C	0.9600
C11—H11C	0.9600	C25—H25A	0.9600
C12—H12A	0.9600	C25—H25B	0.9600
C12—H12B	0.9600	C25—H25C	0.9600
C12—H12C	0.9600	C26—H26A	0.9600
C13—H13A	0.9600	C26—H26B	0.9600
C13—H13B	0.9600	C26—H26C	0.9600
C13—H13C	0.9600		
			
I1···O7^i^	3.300 (3)	I2···O3^iii^	3.264 (3)
			
C4—I1—O7^i^	160.08 (15)	H13B—C13—H13C	109.5
O4—S1—C1	105.7 (2)	C17—I2—O3^iii^	174.78 (14)
O4—S1—C13	106.0 (3)	O8—S2—C14	107.7 (2)
C1—S1—C13	97.4 (2)	O8—S2—C26	105.5 (2)
O4—S1—O4^ii^	78.94 (17)	C14—S2—C26	97.6 (2)
C1—S1—O4^ii^	174.59 (17)	C21—O5—C20	106.4 (3)
C13—S1—O4^ii^	78.48 (18)	C23—O6—C24	115.7 (4)
C7—O1—C8	106.4 (3)	C21—C14—C15	107.9 (4)
C10—O2—C11	116.0 (4)	C21—C14—S2	122.2 (3)
C8—C1—C2	108.3 (4)	C15—C14—S2	130.0 (3)
C8—C1—S1	123.4 (4)	C20—C15—C16	118.7 (4)
C2—C1—S1	128.3 (3)	C20—C15—C14	104.4 (4)
C3—C2—C7	119.1 (4)	C16—C15—C14	136.8 (4)
C3—C2—C1	136.7 (4)	C17—C16—C15	117.0 (4)
C7—C2—C1	104.2 (4)	C17—C16—H16	121.5
C4—C3—C2	116.8 (4)	C15—C16—H16	121.5
C4—C3—H3	121.6	C16—C17—C18	122.7 (4)
C2—C3—H3	121.6	C16—C17—I2	119.5 (3)
C3—C4—C5	123.2 (4)	C18—C17—I2	117.8 (3)
C3—C4—I1	119.8 (3)	C19—C18—C17	121.4 (4)
C5—C4—I1	117.1 (3)	C19—C18—H18	119.3
C4—C5—C6	120.7 (4)	C17—C18—H18	119.3
C4—C5—H5	119.6	C18—C19—C20	115.2 (4)
C6—C5—H5	119.6	C18—C19—C25	123.8 (4)
C7—C6—C5	115.5 (4)	C20—C19—C25	121.0 (5)
C7—C6—C12	122.6 (5)	O5—C20—C19	124.6 (4)
C5—C6—C12	121.8 (5)	O5—C20—C15	110.5 (4)
C6—C7—O1	124.8 (4)	C19—C20—C15	124.9 (4)
C6—C7—C2	124.6 (4)	C14—C21—O5	110.8 (4)
O1—C7—C2	110.6 (4)	C14—C21—C22	134.1 (4)
C1—C8—O1	110.5 (4)	O5—C21—C22	115.1 (4)
C1—C8—C9	133.7 (5)	C21—C22—C23	114.2 (4)
O1—C8—C9	115.8 (4)	C21—C22—H22A	108.7
C8—C9—C10	113.7 (4)	C23—C22—H22A	108.7
C8—C9—H9A	108.8	C21—C22—H22B	108.7
C10—C9—H9A	108.8	C23—C22—H22B	108.7
C8—C9—H9B	108.8	H22A—C22—H22B	107.6
C10—C9—H9B	108.8	O7—C23—O6	125.1 (4)
H9A—C9—H9B	107.7	O7—C23—C22	126.0 (4)
O3—C10—O2	124.9 (4)	O6—C23—C22	108.8 (4)
O3—C10—C9	124.9 (4)	O6—C24—H24A	109.5
O2—C10—C9	110.2 (4)	O6—C24—H24B	109.5
O2—C11—H11A	109.5	H24A—C24—H24B	109.5
O2—C11—H11B	109.5	O6—C24—H24C	109.5
H11A—C11—H11B	109.5	H24A—C24—H24C	109.5
O2—C11—H11C	109.5	H24B—C24—H24C	109.5
H11A—C11—H11C	109.5	C19—C25—H25A	109.5
H11B—C11—H11C	109.5	C19—C25—H25B	109.5
C6—C12—H12A	109.5	H25A—C25—H25B	109.5
C6—C12—H12B	109.5	C19—C25—H25C	109.5
H12A—C12—H12B	109.5	H25A—C25—H25C	109.5
C6—C12—H12C	109.5	H25B—C25—H25C	109.5
H12A—C12—H12C	109.5	S2—C26—H26A	109.5
H12B—C12—H12C	109.5	S2—C26—H26B	109.5
S1—C13—H13A	109.5	H26A—C26—H26B	109.5
S1—C13—H13B	109.5	S2—C26—H26C	109.5
H13A—C13—H13B	109.5	H26A—C26—H26C	109.5
S1—C13—H13C	109.5	H26B—C26—H26C	109.5
H13A—C13—H13C	109.5		
			
O4—S1—C1—C8	−146.0 (4)	C8—C9—C10—O2	−155.0 (4)
C13—S1—C1—C8	105.1 (4)	O8—S2—C14—C21	−133.1 (4)
O4—S1—C1—C2	35.0 (5)	C26—S2—C14—C21	117.9 (4)
C13—S1—C1—C2	−74.0 (5)	O8—S2—C14—C15	45.9 (5)
C8—C1—C2—C3	176.9 (5)	C26—S2—C14—C15	−63.1 (5)
S1—C1—C2—C3	−4.0 (8)	C21—C14—C15—C20	−0.5 (5)
C8—C1—C2—C7	−2.0 (5)	S2—C14—C15—C20	−179.6 (4)
S1—C1—C2—C7	177.2 (3)	C21—C14—C15—C16	176.6 (5)
C7—C2—C3—C4	−2.1 (6)	S2—C14—C15—C16	−2.5 (8)
C1—C2—C3—C4	179.2 (5)	C20—C15—C16—C17	−0.1 (6)
C2—C3—C4—C5	0.1 (7)	C14—C15—C16—C17	−176.9 (5)
C2—C3—C4—I1	179.4 (3)	C15—C16—C17—C18	−0.9 (7)
O7^i^—I1—C4—C3	150.8 (3)	C15—C16—C17—I2	178.1 (3)
O7^i^—I1—C4—C5	−29.9 (6)	C16—C17—C18—C19	1.1 (8)
C3—C4—C5—C6	1.0 (7)	I2—C17—C18—C19	−177.9 (4)
I1—C4—C5—C6	−178.2 (3)	C17—C18—C19—C20	−0.3 (7)
C4—C5—C6—C7	−0.1 (6)	C17—C18—C19—C25	179.0 (5)
C4—C5—C6—C12	179.5 (4)	C21—O5—C20—C19	−177.8 (5)
C5—C6—C7—O1	179.3 (4)	C21—O5—C20—C15	1.7 (5)
C12—C6—C7—O1	−0.3 (7)	C18—C19—C20—O5	178.6 (4)
C5—C6—C7—C2	−2.0 (7)	C25—C19—C20—O5	−0.6 (8)
C12—C6—C7—C2	178.4 (4)	C18—C19—C20—C15	−0.7 (8)
C8—O1—C7—C6	179.0 (4)	C25—C19—C20—C15	−180.0 (5)
C8—O1—C7—C2	0.1 (5)	C16—C15—C20—O5	−178.5 (4)
C3—C2—C7—C6	3.2 (7)	C14—C15—C20—O5	−0.7 (5)
C1—C2—C7—C6	−177.7 (4)	C16—C15—C20—C19	0.9 (7)
C3—C2—C7—O1	−178.0 (4)	C14—C15—C20—C19	178.7 (5)
C1—C2—C7—O1	1.1 (5)	C15—C14—C21—O5	1.6 (5)
C2—C1—C8—O1	2.2 (5)	S2—C14—C21—O5	−179.2 (3)
S1—C1—C8—O1	−177.0 (3)	C15—C14—C21—C22	−176.3 (5)
C2—C1—C8—C9	−178.5 (5)	S2—C14—C21—C22	2.9 (7)
S1—C1—C8—C9	2.3 (8)	C20—O5—C21—C14	−2.0 (5)
C7—O1—C8—C1	−1.4 (5)	C20—O5—C21—C22	176.3 (4)
C7—O1—C8—C9	179.1 (4)	C14—C21—C22—C23	−68.7 (7)
C1—C8—C9—C10	−92.0 (6)	O5—C21—C22—C23	113.6 (4)
O1—C8—C9—C10	87.3 (5)	C24—O6—C23—O7	1.9 (7)
C11—O2—C10—O3	0.4 (7)	C24—O6—C23—C22	−176.7 (4)
C11—O2—C10—C9	−177.9 (4)	C21—C22—C23—O7	15.9 (7)
C8—C9—C10—O3	26.6 (7)	C21—C22—C23—O6	−165.5 (4)

Symmetry codes: (i) *x*, *y*−1, *z*+1; (ii) −*x*+2, −*y*+1, −*z*+1; (iii) *x*+1, *y*, *z*.

### Hydrogen-bond geometry (Å, °)

**Table:**

*D*—H···*A*	*D*—H	H···*A*	*D*···*A*	*D*—H···*A*
C5—H5···O3^iv^	0.93	2.59	3.430 (6)	150
C11—H11B···O8	0.96	2.51	3.254 (6)	134
C12—H12C···O4^v^	0.96	2.58	3.489 (6)	158
C16—H16···O8^vi^	0.93	2.48	3.373 (6)	160
C18—H18···O4^ii^	0.93	2.44	3.297 (6)	153
C22—H22B···O8^vii^	0.97	2.27	3.215 (6)	164
C12—H12A···Cg1^iv^	0.96	2.85	3.734 (7)	153
C24—H24A···Cg3^viii^	0.96	2.73	3.574 (7)	147

Symmetry codes: (iv) −*x*+1, −*y*, −*z*+1; (v) −*x*+2, −*y*, −*z*+1; (vi) −*x*+2, −*y*+1, −*z*; (ii) −*x*+2, −*y*+1, −*z*+1; (vii) −*x*+1, −*y*+1, −*z*; (viii) −*x*+1, −*y*+2, −*z*.
